# Impact of Coronavirus and Covid-19 on Present and Future Anesthesiology Practices

**DOI:** 10.3389/fmed.2020.00452

**Published:** 2020-07-21

**Authors:** Lingzhong Meng, David L. McDonagh

**Affiliations:** ^1^Department of Anesthesiology, Yale University School of Medicine, New Haven, CT, United States; ^2^Department of Anesthesiology and Pain Management, University of Texas Southwestern Medical Center, Dallas, TX, United States

**Keywords:** coronavirus, Covid-19, pandemic, anesthesia practice, impact, lesson, preparedness, future

## Introduction

**Figure 1:**
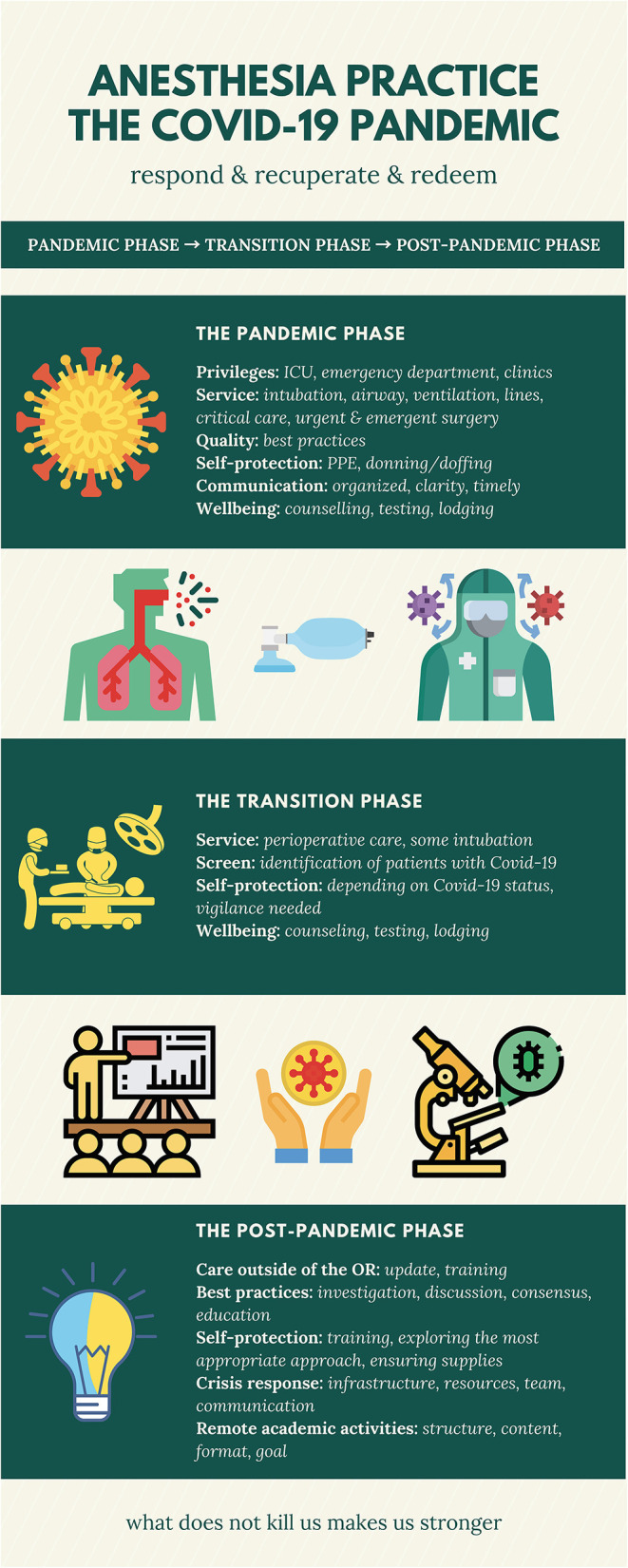
Impact of the Covid-19 pandemic on anesthesia practice through different phases.

The Covid-19 pandemic has swept the world in fewer than 3 months, and there remains no end in sight. Approximately 6.1% of Covid-19 cases were classified as critical—defined as respiratory failure, shock, and multiple organ dysfunction or failure (1). Among the critically ill Covid-19 patients, ~6–47% of them were intubated in China (2–7), 71–75% were intubated in the United States (8, 9), and 88% were intubated in Italy (10). The sheer volume of patients who require invasive mechanical ventilation support entails that anesthesia professionals have been put under significant pressure during this pandemic. This pressure is exacerbated by the fact that many urgent and emergent surgeries must proceed, even in situations in which patients have confirmed or suspected Covid-19. Clearly, anesthesia providers are playing a fundamental role in the frontline efforts to fight against this formidable pandemic. This paper discusses the impact Covid-19 is having on contemporary anesthesia practice through different phases and highlights some of the lessons we can learn to inform future practice (Figure 1).

## The Pandemic Phase

### Role Changes and Issues Identified

Be it as a measure of precaution, resource-saving, better manpower allocation, or ensuring availability of hospital beds, many hospitals throughout the world have canceled or postponed elective and semi-elective surgeries amid the current pandemic. While the reduction in the volume of surgical procedures being performed varies across different hospitals, it can be as high as 70–90%. This move suddenly relieves most anesthesia providers from perioperative care, with only a small portion being deployed to provide anesthesia for urgent or emergent surgeries. At the same time, as a result of the rapidly expanding number of patients admitted to hospitals and intensive care units (ICUs), anesthesiologists are being mobilized and re-deployed to serve outside the perioperative setting.

During this pandemic, anesthesia providers are typically being asked to provide the following services: (1) to intubate critically ill patients who require invasive mechanical ventilatory support; (2) to work in the ICU in the roles of intensivists, respiratory therapists, or nurses; (3) to place intra-arterial catheters and peripheral or central intravenous catheters; and (4) to work in the emergency departments or fever clinics to ensure the gaps in resources created by the sudden increase in symptomatic patients are filled (11). This is the overall global picture; however, the type and load of the work assigned to anesthesia providers outside the perioperative environment primarily depend on the number of cases encountered by individual hospitals and vary by country.

Various issues that directly impact anesthesia providers have arisen in the midst of providing care to critically ill Covid-19 patients. These issues are related to self-protection, best practices of intubation and ventilation, and professional liability in delivering care to patients outside any specialist scope of practice.

### Issue 1: What Are the Most Appropriate Self-Protection Measures?

In mid-March 2020, an article was published documenting the intubation and ventilation experiences in one of the epicenters—Wuhan, China (11). In this paper, the authors described the personal protective equipment (PPE) used by the Chinese healthcare workers. Of note, when performing invasive procedures in Covid-19 patients, including intubation and ventilation, all healthcare workers in China were required to follow Level III protective measures. Put simply, this mandates coverage of the entire body (11). This practice has caused a wide-range discussion outside China. In comparison, in the United States, standard protective practice does not involve covering the neck or leg below the knee. Although we agree that neither under-protection nor over-protection are warranted, the most ideal approach to self-protection is unclear. We hope this information will come to light with future analyses of worldwide practice data.

### Issue 2: How Do We Deal With the Shortage of Personal Protective Equipment?

Regardless of what level of protection is most efficient, the shortage of PPE has caused some significant concerns. Especially at the early stage of the pandemic, there is a global shortage of almost every piece of PPE that is deemed necessary when performing invasive procedures in Covid-19 patients. Many medical practitioners are scrambling to identify methods of sterilizing and reusing N95 masks and/or making their own face shields. Reports of doctors and nurses using unconventional self-protection innovations, such as transparent plastic bags to cover the head and neck, have flooded social media and newspapers. The shortage of PPE and the difference in the availability of self-protection resources across different hospitals, regions, and countries have caused concern and confusion, and this has even resulted in some providers refraining from attending work (12). Moving forward, ensuring adequate PPE supply at all times with a robust production and supply chain capability is a priority.

### Issue 3: What Are the Best Practices for Intubation and Ventilation?

In regards to the best practice when intubating and ventilating Covid-19 patients, there is no universal agreement, but the experiences of different countries should be considered (11, 13–16). Most anesthesia providers typically perform the following steps during intubation: (1) maintain the oxygenation and ventilatory support that has already been used in the patient; (2) avoid bag-mask ventilation if possible; (3) use 100% oxygen for 5 min during pre-oxygenation; (4) cover the patient's nose, mouth, and face; (5) perform rapid sequence induction; (6) aim for complete muscle relaxation; (7) avoid coughing and bucking; (8) perform video laryngoscope guided intubation; and (9) avoid chest auscultation. When delivering ventilatory support, most providers adhere to the following processes. They should avoid non-invasive ventilation, including continuous positive airway pressure and bilevel positive airway pressure, if there are enough ventilators and manpower for invasive mechanical ventilation. This is supported by reports from Lombardy region, Italy, where 11% of cases received non-invasive ventilation and 88% invasive mechanical ventilation during the first 24 h of ICU admission;(10) and in California, United States, 4% of ICU cases received high-flow nasal cannula, 1% non-invasive ventilation, and 91% invasive mechanical ventilation (17). They should adopt lung-protective ventilation strategies; set an ideal oxygenation goal; deliver early prone position ventilation; ensure adequate sedation and analgesia; and provide muscle relaxation when needed (11, 18). Lastly, the best approach to extubation is equally important as it may generate infectious aerosols as a result of patient coughing, and agitation (11).

### Issue 4: How to Protect Anesthesiologists From Liability?

Most anesthesia providers are not credentialled to work outside the perioperative environment, especially in the United States. Although it appears that the Covid-19 crisis is a scenario in which the Good Samaritan principle would apply, there is still a requirement to rapidly authorize anesthesia providers to care for patients in the ICUs, emergency departments, and clinics. Depending on the local policy and practice, credentialing committees should quickly facilitate the process to legally authorize anesthesia providers to deliver necessary services in settings outside the perioperative environment as appropriate.

### Issue 5: How Do We Effectively Organize and Communicate?

The Covid-19 pandemic presents some unprecedented challenges to anesthesiology departments. The environment is sporadic, chaotic, and unpredictable, with the situation changing daily, if not hourly, especially at the early stage of the pandemic. While every effort is made to ensure all practitioners are updated on the current status via timely communications, confusion and anxiety are commonplace. While it is understandable that almost all practitioners are witnessing a crisis of this severity for the first time in their lives, it is imperative that efforts are invested in streamlining the communication process so things proceed in the most smooth and effective fashion (19).

Most anesthesiology departments have quickly established a task force that is specifically responsible for dealing with the Covid-19 crisis. Organized, centralized, clear, and timely communication is essential. The leader of this task force or the individual to whom the leader delegates responsibility needs be in charge of the departmental communication. The message needs be as clear and transparent as possible to avoid any confusion. Reports from front-line staff go to the task force, not the entire department, for collection, summary, and dissemination. Daily conference calls with clinical leadership serve to keep everyone informed and delivering a consistent message to their teams.

### Issue 6: What Are the Measures to Support Anesthesia Providers?

Every effort needs be made to protect frontline providers (19). The anesthesiology department needs work aggressively with hospital partners to seek alternative sources of supplies when facing a shortage of critical PPE and medications. Counseling for mental health and wellbeing needs be provided to department members (20). Lodging can be considered for individuals who are particularly concerned about risks of contamination of their home environment. Departmental leaders are role models for the team members by offering courage, acting as a source of inspiration, and encouraging a spirit of caring for each other.

## The Transition Phase

As of mid-April 2020, the current pandemic appears starting to head into a transition phase, with the progress varying from country to country. The transition phase is characterized by a dramatic decrease, but not complete elimination, of cases and risks of infection. During this phase, regular work order is gradually resumed while continuing to care for varying numbers of Covid-19 patients.

### Issue 1: How to Reopen the Operating Rooms?

During the transition phase, it may be tempting to maximize the capacity of the operating rooms to address the cases that were postponed or rescheduled at the height of the pandemic. However, it is prudent to open the operating rooms more gradually for several reasons. First, the infection risk is lower but still lingers. Infection control requires time and energy and consumes resources. The need to ensure adequate protection and maintain control over the virus should be treated as a higher priority than maximizing caseload, given the potential for severe unintended consequences. Second, perioperative personnel, including anesthesia providers, have relearned and redesigned their approach to patient care to emphasize caution over throughput. Short of a vaccine that abolishes the future risk of Covid-19, there cannot be an immediate return to business as usual. Practitioners likely cannot achieve the necessary level of caution from an infection prevention standpoint, while achieving high throughput surgical volume, without neglecting other aspects of patient care and safety. Third, as we have learned now, some critically ill patients will continue to occupy the ICU beds, even weeks into the transition phase. Therefore, if a surgical case would typically require ICU admission after surgery, there will be a need to coordinate resource management with the hospital bed flow management team. Finally, the spread of the disease (hopefully through community spread and not at-work exposure/infection) will reduce the available workforce unpredictably. Contact tracing and temporary quarantine further reduce the numbers of available workers.

### Issue 2: How Do We Prepare Patients for Surgery?

Vigilance is needed as the risk of infection still exists during the transition phase. It has been suggested that all surgical patients undergo SARS-CoV-2 nucleic acid (typically a PCR test) and antibody tests and chest x-rays or CTs even if they are clinically asymptomatic. False negative rates are non-zero but poorly defined (2–29% estimated based on current data June 2020) (21). The preoperative preparation during the transition phase requires standardized approaches and policies. At a minimum, anesthesia providers need to be cautious during preoperative patient preparation. It is prudent to do the following: (1) wear a surgical mask and eye protection (goggles or face shield) when visiting, interviewing, and examining patients; (2) wash hands before and after each visit; (3) wear gloves when touching and examining patients; (4) consider avoiding chest auscultation if not clinically indicated; (4) be vigilant for the signs of infection; (5) follow up on pertinent labs; (6) follow up on chest x-ray or computed tomography results if ordered; (7) always remember to screen the patient for a history of Covid-19 and/or close contact with confirmed cases; and (8) consider testing for Covid-19 and the presence of an antibody response. Moreover, data regarding the protection provided by an immune response to prior Covid-19 infection and the duration of immunity are desperately needed.

### Issue 3: Protections in the Operating Rooms?

In the operating rooms, full self-protection including N95 masks or power air purifying respirators (PAPRs), goggles or face shields, and waterproof gowns needs be worn if the patient has confirmed or suspected Covid-19; otherwise, wearing a surgical mask with a face shield should be the minimum for patients without evidence of Covid-19. If a Covid-19 patient is undergoing surgery, the following recommendations are advised: 1) perform the surgery in a dedicated Covid-19 negative-pressure operating room; (2) follow the consensus PPE guidelines during intubation and ventilation; (3) ensure smooth emergence and extubation; (4) use filters that are capable of preventing virus transmission/contamination to the anesthesia machine; (5) try to use disposable supplies when possible; and (6) thoroughly clean/sterilize any non-disposable equipment after surgery.

## The Post-pandemic Phase

Even after the pandemic has been officially declared over, things will not go back to how they used to be (even if there is an effective vaccine). The impact of Covid-19 on anesthesia practice will be deeply embedded. As the adage goes: what does not kill us makes us stronger. The lessons that can be learned from this pandemic are summarized below.

### Lesson 1: Infection Control and Prevention

The most effective methods of protecting providers against virus transmission need to be identified (22–24). Different hospitals, regions, and countries have adopted different approaches. Evidence regarding the relationship between the various self-protection mandates that are available and the risk of cross-contamination is needed; neither under-protection nor over-protection is warranted. Different viruses have different behaviors, virulence, and modes of transmission; therefore, preparedness to adjust the approach to self-protection when confronting a novel virus and a new outbreak will be needed. A related issue concerns the adequacy of PPE supplies. Regular stockpile checking needs to be mandated. Methods of sterilizing and reusing different components of PPE need to be investigated and established. The supply chain needs to be bolstered, with contacts regularly maintained. All providers should be trained on the appropriate use of PPE, including the donning and doffing processes.

### Lesson 2: Best Practices of Intubation and Ventilation

The best practices regarding intubation and ventilation need to be elucidated. Although there is some consensus, most of the actions that have been taken thus far amid this pandemic are opinion-based. Evidence to support or revise these is needed. One example is the non-invasive ventilatory support in critically ill Covid-19 patients. Bilevel positive airway pressure ventilation support was popularly used in the epicenter in Wuhan, China (11). Continuous positive airway pressure ventilation support has been used in the United Kingdom (25). However, non-invasive ventilation support has not been widely recommended for use in both Italy and the United States (10, 17). The three primary factors that determine which one to choose are clinical effectiveness, risk of cross-contamination, and the availability of resources. Clearly, the best practices concerning care for critically ill patients in situations like this pandemic need to be further investigated and discussed.

### Lesson 3: The Skill Set for Future Anesthesiologists

The scopes of the clinical skills that future anesthesia providers should possess need to be clarified. This crisis has taught us that, during pandemics of this nature, anesthetists are not only needed for surgical procedures and airway management but also for work in the ICUs, emergency departments, and clinics. It is plausible to quickly teach practitioners immediately before and during the required activities; however, it would be better if the potential need in any future situation similar to this Covid-19 pandemic is anticipated and our providers are proactively trained so that they possess the skills they may need in an emergency situation. The good news is that the skills required outside the perioperative environment (e.g., ventilatory support) are not something unfamiliar to anesthesia providers, as critical care training is a component of anesthesiology residency in most countries. Therefore, regularly updating knowledge and practicing essential skills can be sufficient to ensure preparedness.

### Lesson 4: Effective Team Response

A mechanism is needed to rally the team when situations similar to this pandemic occur again. This mechanism includes the ability to quickly assemble a task force, identify the available resources, establish a channel for efficient and clear communication, allocate jobs based on the strength and talent of individual team members, deliver counseling to ensure mental health and well-being, and closely collaborate with colleagues from other departments. The goal of this mechanism is to help practitioners efficiently join forces during the fight against a hidden enemy. The success in this great fight against SARS-CoV-2 resides in the resilience of all professionals related to the care for the Covid-19 patients, including nurses and physicians at different levels of training and practices and across different specialties. As we applaud this unprecedented all-out effort, we should also plan further team building to better prepare for any future outbreaks or pandemics.

### Lesson 5: Continuous Academic Efforts

In a crisis like the Covid-19 pandemic, the traditional conduct of education and research are not permissible due to concerns surrounding virus transmission. Many trainees and research personnel have to stay at home for weeks. Instead of staying passive, anesthesia providers should use this period of time to effectively enhance education and research. Doing so also promotes a feeling of enrichment and satisfaction, which is a positive way of promoting well-being. The widely available remote conferencing platforms revolutionize how people are connected with each other in the modern era, making virtual academic activities possible. Contemporary technologies also allow people to gather online, see each other, talk to each other, reconnect with each other, help each other, exchange information, and move forward together as a team. Finally, the coming months during which people are awaiting for a Covid-19 vaccine will hopefully see a true tipping point in the transition to distance learning, expansion of telemedicine, and remote conferencing that will replace destination continuous medical education, non-essential face-to-face patient encounters, and convention center society meetings.

## Summary

The impact of the Covid-19 pandemic on anesthesia practice varies dynamically with the various phases of the pandemic. As we respond, recuperate, and move forward from the Covid-19 pandemic, the impact on anesthesia practice and the lessons learned should be summarized and addressed to ensure better preparedness and results in the future. The areas in which improvements are needed center on self-protection, best practices, scope of practice, organized response, and remote education, research, and gathering. Preparedness may use certain resources and cause financial concern, especially when a crisis is not observed for many years. Therefore, it would be wise to use the process of preparedness to promote a higher quality of patient care, education, research, and culture building. Simulation and quality assurance activities will facilitate “maintenance of preparedness.” *Vigilance* is the motto of the North American anesthesiology community, and it appears to be more appropriate now than ever.

## Author Contributions

LM helped with the concept and design, administrative and material support, data interpretation, manuscript drafting, and critical revision of the manuscript for important intellectual content. DM helped with the data interpretation and critical revision of the manuscript for important intellectual content. All authors contributed to the article and approved the submitted version.

## Conflict of Interest

The authors declare that the research was conducted in the absence of any commercial or financial relationships that could be construed as a potential conflict of interest.

## Abbreviations

Covid-19: coronavirus disease 2019
ICU: intensive care unit
PPE: personal protective equipment.

## References

[1] Phua J Weng L Ling L Egi M Lim CM Divatia JV. Intensive care management of coronavirus disease 2019 (COVID-19): challenges and recommendations. Lancet Respir Med. (2020) 8:506–17. 10.1016/S2213-2600(20)30161-2 32272080 PMC7198848

[2] Huang C Wang Y Li X Ren L Zhao J Hu Y. Clinical features of patients infected with 2019 novel coronavirus in Wuhan, China. Lancet. (2020) 395:497–506. 10.1016/S0140-6736(20)30183-5 31986264 PMC7159299

[3] Tang X Du R Wang R Cao T Guan L Yang C Comparison of Hospitalized Patients with Acute Respiratory Distress Syndrome Caused by COVID-19 and H1N1. Chest. (2020) 158:195–205. 10.1016/j.chest.2020.03.032 32224074 PMC7151343

[4] Wang D Hu B Hu C Zhu F Liu X Zhang J. Clinical characteristics of 138 hospitalized patients with 2019 novel coronavirus-infected pneumonia in Wuhan, China. JAMA. (2020) 323:1061–9. 10.1001/jama.2020.1585 32031570 PMC7042881

[5] Wang Y Lu X Chen H Chen T Su N Huang F. Clinical Course and Outcomes of 344 Intensive Care Patients with COVID-19. Am J Respir Crit Care Med. (2020) 201:1430–4. 10.1164/rccm.202003-0736LE 32267160 PMC7258632

[6] Wu C Chen X Cai Y Xia Ja Zhou X Xu S. Risk factors associated with acute respiratory distress syndrome and death in patients with coronavirus disease 2019 pneumonia in Wuhan, China. JAMA Intern Med. (2020) 180:934–3. 10.1001/jamainternmed.2020.0994 32167524 PMC7070509

[7] Yang X Yu Y Xu J Shu H Xia Ja Liu H. Clinical course and outcomes of critically ill patients with SARS-CoV-2 pneumonia in Wuhan, China: a single-centered, retrospective, observational study. Lancet Respir Med. (2020) 8:475–81. 10.1016/S2213-2600(20)30079-5 32105632 PMC7102538

[8] Arentz M Yim E Klaff L Lokhandwala S Riedo FX Chong M. Characteristics and Outcomes of 21 Critically Ill Patients With COVID-19 in Washington State. JAMA. (2020) 323:1612–4. 10.1001/jama.2020.4326 32191259 PMC7082763

[9] Bhatraju PK Ghassemieh BJ Nichols M Kim R Jerome KR Nalla AK. Covid-19 in critically ill patients in the Seattle region - case series. N Engl J Med. (2020) 382:2012–22. 10.1056/NEJMoa2004500 32227758 PMC7143164

[10] Grasselli G Zangrillo A Zanella A Antonelli M Cabrini L Castelli A. Baseline characteristics and outcomes of 1591 patients infected with SARS-CoV-2 admitted to ICUs of the Lombardy Region, Italy. JAMA. (2020) 323:1574–81. 10.1001/jama.2020.5394 32250385 PMC7136855

[11] Meng L Qiu H Wan L Ai Y Xue Z Guo Q. Intubation and ventilation amid the COVID-19 outbreak: Wuhan's experience. Anesthesiology. (2020) 132:1317–32. 10.1097/ALN.0000000000003296 32195705 PMC7155908

[12] Newman M. Covid-19: doctors' leaders warn that staff could quit and may die over lack of protective equipment. BMJ. (2020) 368:m1257. 10.1136/bmj.m1257 32217522

[13] Yao W Wang T Jiang B Gao F Wang L Zheng H. Emergency tracheal intubation in 202 patients with COVID-19 in Wuhan, China: lessons learnt and international expert recommendations. Brit J Anaesthesia. (2020) 125:e28–37. 10.1016/j.bja.2020.03.026 32312571 PMC7151238

[14] Sorbello M El-Boghdadly K Di Giacinto I Cataldo R Esposito C Falcetta S. The Italian coronavirus disease 2019 outbreak: recommendations from clinical practice. Anaesthesia. (2020) 75:724–32. 10.1111/anae.15049 32221973

[15] Cook T El-Boghdadly K McGuire B McNarry A Patel A Higgs A. Consensus guidelines for managing the airway in patients with COVID-19: guidelines from the Difficult Airway Society, the Association of Anaesthetists the Intensive Care Society, the Faculty of Intensive Care Medicine and the Royal College of Anaesthetists. Anaesthesia. (2020) 75:785–99. 10.1111/anae.15054 32221970 PMC7383579

[16] Brewster DJ Chrimes NC Do TB Fraser K Groombridge CJ Higgs A. Consensus statement: safe Airway Society principles of airway management and tracheal intubation specific to the COVID-19 adult patient group. Med J Aust. (2020) 16:472–81. 10.5694/mja2.50598 32356900 PMC7267410

[17] Myers LC Parodi SM Escobar GJ Liu VX. Characteristics of Hospitalized Adults With COVID-19 in an Integrated Health Care System in California. JAMA. (2020) 323:2195–8. 10.1001/jama.2020.7202 32329797 PMC7182961

[18] Alhazzani W Møller MH Arabi YM Loeb M Gong MN Fan E. Surviving Sepsis Campaign: guidelines on the management of critically ill adults with Coronavirus Disease 2019 (COVID-19). Intensive Care Med. (2020) 48:e440–69. 10.1097/CCM.0000000000004363 32222812 PMC7101866

[19] Adams JG Walls RM. Supporting the health care workforce during the COVID-19 global epidemic. JAMA. (2020) 323:1439–40. 10.1001/jama.2020.3972 32163102

[20] Chen Q Liang M Li Y Guo J Fei D Wang L. Mental health care for medical staff in China during the COVID-19 outbreak. Lancet Psychiatry. (2020) 7:e15–6. 10.1016/S2215-0366(20)30078-X 32085839 PMC7129426

[21] Woloshin S Patel N Kesselheim AS. False negative tests for SARS-CoV-2 infection—challenges and implications. N Engl J Med. (2020). 10.1056/NEJMp2015897. [Epub ahead of print]. 32502334

[22] Bowdle A Munoz-Price LS. Preventing infection of patients and healthcare workers should be the new normal in the era of novel coronavirus epidemics. Anesthesiol J Am Soc Anesthesiologists. (2020) 132:1292–5. 10.1097/ALN.0000000000003295 32195701 PMC7155906

[23] Wax RS Christian MD. Practical recommendations for critical care and anesthesiology teams caring for novel coronavirus (2019-nCoV) patients. Can J Anesthesia. (2020) 67:568–76. 10.1007/s12630-020-01591-x 32052373 PMC7091420

[24] Tran K Cimon K Severn M Pessoa-Silva CL Conly J. Aerosol generating procedures and risk of transmission of acute respiratory infections to healthcare workers: a systematic review. PLoS ONE. (2012) 7:e35797. 10.1371/journal.pone.0035797 22563403 PMC3338532

[25] Baker JG Sovani M. Case for continuing community NIV and CPAP during the COVID-19 epidemic. Thorax. (2020) 75:368. 10.1136/thoraxjnl-2020-214913 32273336

